# Prevalence of osteoporosis and incidence of hip fracture in women - secular trends over 30 years

**DOI:** 10.1186/1471-2474-11-48

**Published:** 2010-03-11

**Authors:** Henrik G Ahlborg, Björn E Rosengren, Teppo LN Järvinen, Cecilia Rogmark, Jan-Åke Nilsson, Ingemar Sernbo, Magnus K Karlsson

**Affiliations:** 1 Clinical and Molecular Osteoporosis Research Unit, Department of Clinical Science, Lund University, Malmö University Hospital, SE-20502 Malmö, Sweden; 2 Department of Orthopaedics and Traumatology, Tampere University Hospital, Tampere, Finland

## Abstract

**Background:**

The number of hip fractures during recent decades has been reported to be increasing, partly because of an increasing proportion of elderly women in the society. However, whether changes in hip fracture annual incidence in women are attributable to secular changes in the prevalence of osteoporosis is unclear.

**Methods:**

Bone mineral density was evaluated by single-photon absorptiometry at the distal radius in 456 women aged 50 years or above and living in the same city. The measurements were obtained by the same densitometer during three separate time periods: 1970-74 (n = 106), 1987-93 (n = 175) and 1998-1999 (n = 178), and the age-adjusted prevalence of osteoporosis in these three cohorts was calculated. Additionally, all hip fractures sustained in the target population of women aged 50 years or above between 1967 and 2001 were registered, whereupon the crude and the age-adjusted annual incidence of hip fractures were calculated.

**Results:**

There was no significant difference in the age-adjusted prevalence of osteoporosis when the three cohorts were compared (P = 1.00). The crude annual incidence (per 10,000 women) of hip fracture in the target population increased by 110% from 40 in 1967 to 84 in 2001. The overall trend in the crude incidence between 1967 and 2001 was increasing (1.58 per 10,000 women per year; 95 percent confidence interval, 1.17 to 1.99), whereas the age-adjusted incidence was stable over the same period (0.22 per 10,000 women per year; 95 percent confidence interval, -0.16 to 0.60).

**Conclusions:**

The increased number of hip fracture in elderly women is more likely to be attributable to demographic changes in the population than to secular increase in the prevalence of osteoporosis.

## Background

Hip fracture represents the hallmark of the fracture epidemic among elderly people, because of the associated mortality, morbidity, and health care costs [1,2]. The annual incidence of hip fractures has been reported to be on the increase worldwide during the last five decades, [3-12] so that the current total number of women sustaining a hip fracture is estimated at one million annually [13]. Although the increase in hip fractures at least partly could be attributed to demographic changes, i.e. an increasing proportion of elderly women in the population, the specific reason for the reported concomitant increase in the age-adjusted annual incidence of hip fracture during the same time period [3,7,9,14] remains unresolved. For example in Finland, the age-adjusted annual incidence of hip fractures in women aged 50 years or above increased by 60% between 1970 and 1997 [7], and has since been shown to level off [15], in agreement with reports from other regions showing a stabilising pattern [5,14-17]. However, the specific reason for the observed secular patterns in hip fracture incidence during the last decades has not been thoroughly investigated.

In addition to the obvious paramount role of advancing age on the occurrence of hip fractures, both skeletal and non-skeletal risk factors have been identified. According to the conventional prevailing view, low bone mineral density is considered to be the major risk factor for hip fractures [18-20]. The risk of hip fracture more than doubles for each SD lower bone mineral density, and about half of the elderly women with a hip fracture have osteoporosis, i.e. a bone mineral density that is 2.5 SD below the mean of the young population [21]. Although the prevalence of osteoporosis has been characterised in several populations [22-26], to our knowledge, no study has investigated long-term secular patterns in the prevalence of osteoporosis, or addressed the question whether the increased incidence of hip fracture during the last few decades could be attributable not only to demographic changes in the population but also to an increased prevalence of osteoporosis.

This study was therefore designed to characterise secular patterns in the prevalence of osteoporosis and the incidence of hip fracture within the same female target population during the last three decades.

## Methods

### Target population

Demographic data concerning the target population were drawn from the Yearbook of Local Statistics, which includes the annual number of inhabitants in the city of Malmo, Sweden, by gender and one-year age groups. In 1967, the female population in Malmo was 134,586. During the 34-year period 1967 to 2001, the female population increased by 0.6 percent, whereas the percentage of women aged 50 years or above increased from 35 to 39 percent, and the number of women aged 85 years or above increased from 1,499 in 1967 to 5,646 in 2001.

### Study group for bone mineral density measures

We included bone mineral density measures collected between 1970 and 1999 from three different normative cohorts of women aged 50 to 90 years from the target population. The study group consisted of 456 women measured during three different time periods: (1) 106 women were measured between 1970 and 1974 [27]; (2) 175 women between 1987 and 1993 [28,29]; and (3) 178 women were measured between 1998 and 1999 [30]. The subjects in the first two samples were recruited in the same manner: by asking hospital personnel and their relatives, and visitors to hospital in-patients, whether they would participate in a bone mineral study. All were without known metabolic disease or other conditions known or suspected to interact with the bone mineral density. The third cohort was population-based. An invitation letter was sent to 273 women randomly selected from the National Population Records. The attendance rate was 65%. Characteristics of the study group are presented in Table 1 and Table 2.

**Table 1:** Age distribution of the study participants subjected to bone mass measurements in three cohorts of women measured in 1970-74, 1987-93 and 1998-99.

Age class (years)	Cohort 1970-74	Cohort 1987-93	Cohort 1998-99
	n = 106	n = 175	n = 178
Age 50-59	24	49	53
Age 60-69	40	46	41
Age 70-79	28	47	41
Age 80-90	14	33	43

**Table 2:** Characteristics of the study participants subjected to bone mass measurements in three cohorts of women measured in 1970-74, 1987-93 and 1998-99.^a^

Characteristic	Cohort 1970-74	Cohort 1987-93	Cohort 1998-99	***P***- **value**^**c**^
	n = 106	n = 175	n = 178	
Age (yr)	67 (9.5)	68 (10.8)	65 (12.2)	0.01
Height (cm)	163 (6.0)	162 (5.8)	162 (6.9)	0.97
Weight (kg)	67 (11.0)	66 (12.2)	69 (12.5)	0.08
Body-mass index^b^	25 (3.9)	25 (4.4)	26 (4.7)	0.08

^a ^Values are means(SD).

^b ^The body-mass index is the weight in kilograms divided by the square of the height in metres.

^c ^Comparing all three cohorts by analysis of variance.

### Measurement of bone mineral density

Bone mineral density (mg/cm^2^) of the forearm was measured at a site 6 cm proximally of the ulnar styloid process by single-photon absorptiometry according to a protocol described in detail previously [31]. The precision of the method, measured as the week-to-week variation over one year, estimated by standardized phantom data, amounted to slightly less than 1% (coefficient of variation), which corresponds to the stochastic nature of the radiation and also to the geometrical uncertainty in the positioning of the phantom. The reproducibility *in vivo*, determined by duplicate measurements in 20 individuals measured on two occasions weeks or months apart, amounted to 4% (coefficient of variation). The calculation of reproducibility *in vivo *includes the assumption that the bone mineral density is unchanged between the two separate measurements. In this study the same densitometer was used throughout the study, and during the follow-up repeated measurements of a standardized phantom were undertaken every second week. The precision of the method measured as year-to-year variation during the follow-up, estimated by the phantom data, was for bone mineral density 1.8% (coefficient of variation). To determine whether there was any long-term drift of the densitometer during the follow-up, all phantom data were analyzed using a linear regression equation. This analysis showed no significant long-term drift of the equipment 0.1%/year (R^2^<1%; 95% CI -0.2 to 0.4) [32]. Because of the replacement of the radiation source in 1980, all measurements thereafter were adjusted with the use of the data from the phantom.

### Definition of osteoporosis

The WHO cut-off values for the definition of osteoporosis were used [33]. The cut-off values used in this study were derived from a non-population-based sample of 38 healthy women aged 20 to 39 years, measured at the forearm with the same densitometry equipment as in the present study in 1971. The mean (SD) bone mineral density at the distal measurement site in this young reference population was 542 (76) mg/cm^2^. Osteoporosis was defined as a bone mineral density value less than 2.5 SD below the mean of the young reference population.

### Registration of hip fractures

All fractures of the proximal femur (hip fractures), that occurred in the *target population *of women aged 50 years or older, in the time between 1967-68, 1974-75[34], 1980-85[35], 1987-95[8,36] and 1999-2001 [37], were identified at the Department of Diagnostic Radiology and Department of Orthopaedics. Malmo University Hospital has the only emergency department in Malmo, and it has been estimated that more than 97% of all patients with a fracture sustained in Malmo are seen in its trauma unit[38] Subjects who had hip fractures sustained outside of Malmo were subsequently referred to the Orthopaedic Department for a follow-up visit at which the fracture was classified to ensure complete case ascertainment. Non-residents who were examined at the hospital were identified and excluded. The same fracture registration method was used throughout the observation period [8,34-36].

### Statistical analysis

To compare the bone mineral density in the three different cohorts the analysis of variance was used as well as the analysis of covariance with adjustment for age at examination. The prevalence of osteoporosis in the three cohorts were compared with each other by the chi-squared test as well as with logistic regression analysis, with adjustment for age at examination by direct standardisation. As the age-adjusted prevalence of osteoporosis was found not to differ between the three cohorts, all the data were merged and the age-specific prevalence of osteoporosis was calculated within five-year age classes in women aged 50 years or above. The age-specific prevalence and the demographic data of the target population were then used to estimate the annual prevalence of osteoporosis in the target population during the study period.

The incidence of hip fracture in women aged 50 years or above was calculated and expressed as the annual number of hip fractures per 10,000 women. In the calculation of the age-adjusted fracture incidence, age adjustment was done by direct standardisation with the mean population between 1967 and 2001 as the standard population. Time-trend analysis was done by linear regression analysis. Based on previous findings [8], we selected 1985 *a priori *as the hypothetic year to test whether any trend-break in hip fracture incidence occurred during the study period. The difference between time-trend before and after this time-point was analysed in a regression model with an interaction term. All analyses were performed using the SAS ver 9.1 statistical analysis system (SAS Institute, Cary, NC, USA). A P value of less than 0.05 or a positive lower limit of the confidence interval for the time-trend analysis of hip fracture incidence was considered to indicate statistical significance.

Given the size of the study groups and the distribution of the bone mineral density, posthoc two-tailed power analysis yielded a detection of group differences in bone mineral density of between 22 to 25 mg/cm^2 ^with a significance criterion of 0.05 and 80% power.

### Ethics

At the start of the study in 1970, no permission from the institutional review board and no consent form were required; the women were asked to provide oral informed consent. However, later in the study, in 1998, written permission was granted by the ethics committee of the University of Lund (LU 208-98). The study was carried out in accordance with the Helsinki Declaration.

## Results

### Bone mineral density

The three cohorts of women measured in 1970-1974, 1987-1993 and 1998-1999 did not differ statistically significantly with regard to bone mineral density at the distal radius (P = 0.15, Table 3).

**Table 3:** Bone mineral density and prevalence of osteoporosis evaluated at the distal radius in three cohorts of women measured in 1970-74, 1987-93 and 1998-99.

**Measurements**^**a**^	Cohort 1970-74	Cohort 1987-93	Cohort 1998-99	*P*- value
	n = 106	n = 175	n = 178	
Bone mineral density (mg/cm^2^)^b^				
Non-adjusted	438 (422, 454)	453 (439, 468)	461 (446, 477)	0.15^d^
Age-adjusted	444 (430, 458)	460 (449, 471)	451 (440, 461)	0.17^e^
Prevalence of osteoporosis ^c^				
Non-adjusted	15.1 (8.2, 22.0)	15.1 (9.7, 20.5)	14.9 (9.5, 20.2)	1.00^f^
Age-adjusted	13.6 (6.1, 21.2)	14.7 (8.9, 20.5)	11.0 (8.4, 13.6)	1.00 ^g^

^a ^The bone mineral density in the forearm was measured at a site 6 cm (distal radius) proximal to the styloid process of the ulna by single-photon absorptiometry.

^b ^Values are mean (95 percent confidence interval).

^c ^Values are percentages of valid measurements (95 percent confidence interval). Osteoporosis denotes a bone mineral density 2.5 SD below the bone mineral density in young healthy women.

^d ^Comparing all three cohorts by analysis of variance.

^e ^Comparing all three cohorts by analysis of covariance with adjustment for age.

^f ^Comparing all three cohorts by chi-squared test.

^g ^Comparing all three cohorts, with direct standardisation for age, by logistic regression.

There was a statistically significant difference in age between the three cohorts (P = 0.01, Table 2). After adjustment for age, there also was no significant difference in bone mineral density between the three cohorts (P = 0.17, Table 3).

### Prevalence of osteoporosis

The age-adjusted prevalence of osteoporosis varied between 11 and 15 percent in the women measured in 1970-1974, 1987-1993 and 1998-1999. However, there was no difference in the age-adjusted prevalence of osteoporosis between the three cohorts (P = 1.00, Table 3).

**Figure 1:**
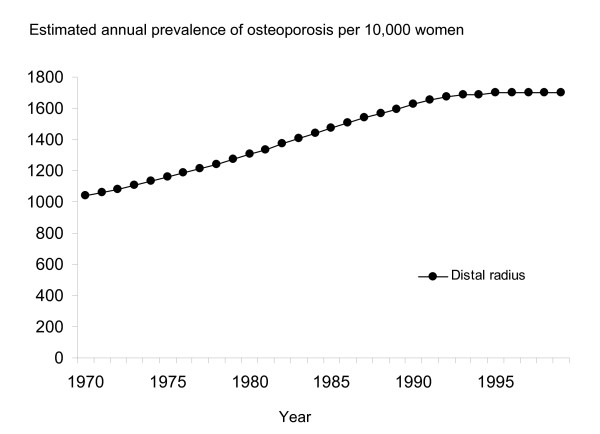
**Estimated prevalence of osteoporosis (per 10,000 women aged 50 years or more) at the distal radius measurement site during the period 1970 to 1999 in Malmö, Sweden**.

As the age-adjusted prevalence did not show a significant change over time, we calculated the age-specific prevalence of osteoporosis and applied these figures to the annual demographic data of the target population to estimate the annual prevalence of osteoporosis in the actual target population. The estimated annual prevalence of osteoporosis in the target population increased by approximately 60% during the study period; from 1,061 per 10,000 women aged 50 years or above in 1970 to 1,698 per 10,000 women aged 50 years or above in 1999 (Figure 1).

### Incidence of hip fractures

**Figure 2:**
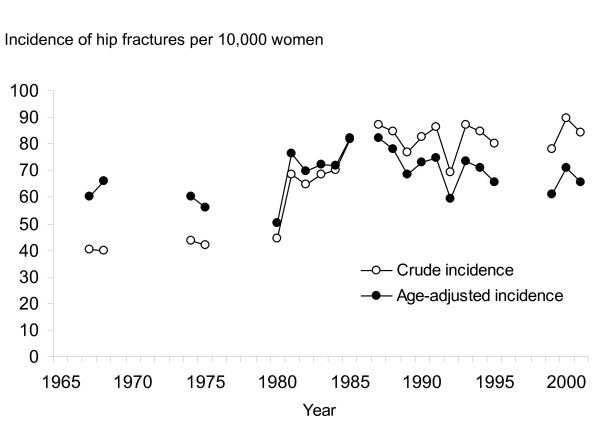
**Crude incidence and age-adjusted incidence (per 10,000 women aged 50 years or more) of hip fractures during the period 1967 to 2001 in Malmö, Sweden**. Incidence rate are only given for the years that fracture data collection were undertaken.

During the 34-year observation period from 1967 to 2001, altogether 1,136,435 person-years were generated and 8,059 hip fractures were registered in the target population of women aged 50 years or above. The annual number of hip fractures increased by 132% from 189 in 1967 to 439 in 2001. As the total number of women aged 50 years or above in the population increased only slightly during the observation period, the crude annual incidence (per 10,000 women aged 50 years or above) of hip fractures increased by 110% from 40 in 1967 to 84 in 2001 (Figure 2).

The overall pattern in the crude incidence between 1967 and 2001 was increasing (1.58 per year; 95 percent confidence interval, 1.17 to 1.99), and was predominantly observed in the time period from 1967 to 1985 (2.09 per year; 95 percent confidence interval, 1.09 to 3.09). Thereafter the incidence levelled off (0.36 per year; 95 percent confidence interval, - 0.46 to 1.19). The difference between the patterns before and after 1985 was statistically significant (P = 0.02, Figure 2).

After adjustment for an increasing proportion of elderly women in the target population, there was no statistically significant change in the age-adjusted incidence of hip fractures over the observation period (0.22 per year; 95 percent confidence interval, - 0.16 to 0.60, Figure 2).

## Discussion

The results of this study show that despite a more than a doubling in the annual number of hip fractures in women aged 50 years or above in the past 30 years in Malmö, Sweden, there was no change in the age-adjusted incidence of hip fractures over the same period. Consistent to the finding of a stable age-adjusted annual incidence of hip fracture a stable age-adjusted bone mineral density as well as a stable prevalence of osteoporosis was observed during the same period. However, when the age-specific prevalences of osteoporosis in the study group were applied to the demographic changes in the whole population of Malmö, calculations suggested that both the total number of women aged 50 years or above with osteoporosis and the annual prevalence of osteoporosis in the target population increased during the observation period. Taken together, these findings suggest that the recent surge in the number of hip fractures of elderly is attributable to demographic changes in the population, i.e. a higher proportion of aged people, rather than to a proposed secular increase in the age-adjusted prevalence of osteoporosis.

Low bone mineral density is considered to be one of the major risk factors for hip fracture [39]. The diagnosis of osteoporosis is also based on a measurement of bone mineral density, and is used for the clinical decision about treatment [33]. The prevalence of osteoporosis has been found to vary considerable between different elderly populations (from 8% to 38%), and between measurement sites within the same population (from 3% to 26%) [22-26]. In this study the age-adjusted prevalence of osteoporosis varied between 11 and 15% during the study period and there was no tendency to any increasing age-adjusted prevalence of osteoporosis in women aged 50 years or above during the evaluated period. However, as the bone mineral density is an age-dependent variable, i.e. the prevalence of osteoporosis increases with advancing age, the age structure of the whole population of women aged 50 years or above must be taken into account when estimating the annual prevalence of osteoporosis in a population. Accordingly, to analyse secular patterns in the annual prevalence of osteoporosis within a society, changes in the age structure must also be taken into account. Although the age-adjusted prevalence in this study was stable over time, the proportion of elderly women increased over time and thus the number of women in the whole population with osteoporosis increased.

The burden of hip fractures has increased considerably throughout the world over the last few decades as both the number and the proportion of elderly women have increased [12]. However, there have been indications of secular deterioration, as the age-adjusted annual incidence of hip fractures has been reported to be increasing in several populations between the 1950s and the 1980s, even after controlling for demographic changes [3,5,7,9,14]. In this study, the rise in the crude incidence levelled off in the mid-1980s, whereas the age-adjusted incidence was stable during the whole observation period. This finding is consistent with studies from the USA (white) and England [5,14,15,17], but contrary to studies from the USA (Hispanics), Singapore and Finland [7,11,14]. Notably, a downturn in hip fracture incidence during the last decade has recently been noted in Finland[15]. However, the reason for the substantial variation in the secular pattern in the age-adjusted incidence between different populations is unclear.

One strength of this study was that the measurements obtained by singe-photon absorptiometry were performed using the same densitometer, without any significant long-term drift, throughout the study period. Furthermore, the system for the ascertainment of hip fractures was the same over the study period [8,34-36], a well-evaluated method that minimised selection bias and misclassification bias, which are usually limiting factors in the use of admission or discharge registers.

However, our study has limitations that require consideration. First, the study group for the bone mineral density measurements were not strictly population-based and thus there is a potentially risk for a selection bias. It could be argued that a non population-based sample consists of healthier individuals and therefore, possibly, the bone mineral density will be overestimated in such a sample. However, the risk for selection bias could not be ruled out even in a population-based sample. A low participation-rate in a population-based sample may overestimate the bone mineral density because the non-responders tend to have a lower bone mineral density than the responders [40]. Second, the WHO diagnostic criteria for osteoporosis includes only postmenopausal women assessed by dual energy X-ray absorptiometry technique, but this measurement equipment was not introduced at the beginning of this study. However, it has been shown that bone mineral density measured at the distal radius by single-photon absorptiometry and at the hip by dual energy X-ray absorptiometry technique are highly correlated (r = 0.9, P < 0.001) [28], and that measurements at the distal radius by single-photon absorptiometry technique also predict hip fractures [41]. It should be noted that even if there is a high correlation between two different measurement techniques there will be a risk of systematic shifts in the data. In this study that could have lead to an under- or overestimation of the prevalence of osteoporosis as compared to if the osteoporosis diagnosis would have been assessed by dual energy X-ray absorptiometry technique. However, as the aim of this study was to evaluate differences and secular patterns in osteoporosis prevalence between three different cohorts, such a systematic shift would not have influenced the results in this study. Moreover, in this study the cut-off values for osteoporosis were derived from a non-population based sample of young women. This may introduce an error in the diagnosis of osteoporosis. It is possible that the mean bone mineral density in a non-population based sample would have been higher than the mean bone mineral density of a population-based sample. Accordingly, this may lead to an underestimation of the prevalence of osteoporosis in this study. However, as the same cut-off values were used in the comparison between the cohorts this would not have influenced the analysis in this study. Third, the relative small numbers of women in each of the three study cohorts lowered the statistical power of this study. However, posthoc power analysis showed that this study had an 80% power to detect a group differences in bone mineral density of between 22 to 25 mg/cm^2^, which corresponds to a group difference of approximate 0.25 SD. As a lowering of one SD in bone mineral density doubles the risk of sustaining a hip fracture, it is reasonable to define the minimal clinically important difference in bone mineral density over three decades and between two different cohorts in such a study as this to 0.25 SD. Given that magnitude of minimal clinically important difference the study samples of the three different cohorts of women in this study were within an acceptable range.

## Conclusion

The observed rise in the number of hip fractures in the elderly female population of Malmo, Sweden, during the last three decades seems to be attributable to an increased proportion of elderly women in the population, and less likely to an increase in the prevalence of osteoporosis.

## Competing interests

The authors declare that they have no competing interests.

## Authors' contributions

Study design: HA, BR, TJ and MK; Data collection: HA, CR, IS, MK; Statistical analysis: HA, BR, JN; Writing the paper: HA, BR, TJ and MK. All authors read and approved the final manuscript.

## Pre-publication history

The pre-publication history for this paper can be accessed here:

http://www.biomedcentral.com/1471-2474/11/48/prepub
